# Changes in accessibility and preferences predict children's future fruit and vegetable intake

**DOI:** 10.1186/1479-5868-2-15

**Published:** 2005-10-10

**Authors:** Elling Bere, Knut-Inge Klepp

**Affiliations:** 1 Department of Nutrition, Faculty of Medicine, University of Oslo, Norway; 2 Department of Nutrition, Box 1046 Blindern, 0316 Oslo, Norway

## Abstract

**Background:**

Most children eat fewer fruits and vegetables than recommended. To be able to design effective interventions, understanding the aetiology of the behaviour is important. Accessibility and preferences have shown to be strong correlates of fruit and vegetable intake in several cross-sectional studies. The aim of this study was to identify predictors of future fruit and vegetable intake and to explore longitudinal patterns of interactions between accessibility and preferences.

**Methods:**

Data presented are based on baseline (September 2001) and follow-up (May/June 2002) surveys of 20 control schools in the Norwegian intervention study *Fruits and Vegetables Make the Marks*. A total of 816 pupils (77%) completed both baseline and follow-up questionnaires. The average age of the sample at baseline was 11.8 years. The research instrument assessing potential predictor variables was guided by Social Cognitive Theory, and included Accessibility at home, Accessibility at school, Modelling, Intention, Preferences, Self-Efficacy and Awareness of the 5-a-day recommendations. Multiple regression analyses were performed.

**Results:**

All independent variables (measured at baseline) were significantly correlated to future fruit and vegetable intake (measured at follow-up). When reported fruit and vegetable intake at baseline (past intake) was included in this model, the effect of the other independent variables diminished. Together with past intake, the observed change in the independent variables from baseline to follow-up explained 43% of the variance in the reported intake at follow-up. Past intake remained the strongest predictor, but changes in accessibility at home and at school, as well as changes in preferences for fruits and vegetables, also explained significant amounts of the variance in fruit and vegetable intake at follow-up. In addition, baseline accessibility was found to moderate the relationship between change in preferences and change in intake.

**Conclusion:**

Change in accessibility and preferences appear to be important predictors of future fruit and vegetable intake among school children. Interventions should focus on strategies to modify these factors.

## Background

Most children eat fewer fruits and vegetables than recommended. To be able to design effective interventions, in order to increase fruit and vegetable consumption, it is critical to understand the aetiology of the behaviour. An intervention should aim at changing the strongest determinants of the behaviour, in order to be successful [1].

Behavioural theories, like Social Cognitive Theory (SCT) [2], provide frameworks for understanding health behaviour and can guide the selection of potential determinants [3]. SCT is extensively used when children's fruit and vegetable intake is the behavioural outcome, and it served as the theoretical framework for four out of five multi-component intervention studies recently reviewed [4].

Several factors have been suggested as determinants of children's fruit and vegetable intake [5]. Among these factors, accessibility and preferences have been most strongly correlated to intake in several studies [6-8]. These studies have, however, been conducted using cross-sectional designs. This is a limitation as cross-sectional relationships could be due to a third antecedent, cannot state causality, and the relationships could be functionally different in longitudinal studies [1]. Longitudinal studies are therefore highly requested in order to prospectively investigate such relationships [1,6,8].

Adolescent fruit and vegetable intake declines with increasing age, but has shown to be stable with respect to the relative intake between individuals [9]. Lien and colleagues [10] reported that the only significant variable in a longitudinal study investigating the variance in fruit and vegetable intake (at age 21) was past intake (at age 15). Eight percent of the variance in fruit and vegetable intake at age 21 was explained by fruit and vegetable intake at age 15 for boys, and 26% (low SES) and 20% (high SES) for girls [10]. Changes in determinants must, however, explain the variance in future fruit and vegetable intake, in addition to the variance explained by past intake, as the change from past to future intake has to be explained. A change in intake is indeed the ultimate goal for an intervention.

The aim of the present study was to identify predictors of future fruit and vegetable intake, to assess whether these factors predicted future fruit and vegetable intake when controlling for past intake, and to assess whether changes in these factors over time were related to future intake and to the change in intake over time. In addition, a secondary aim was to explore longitudinal patterns of interactions between accessibility and preferences.

## Methods

### Sample and procedure

Data presented are based on the baseline (September 2001) and follow-up (May/June 2002) surveys of the 20 control schools in the intervention project *Fruits and Vegetables Make the Marks *(FVMM). These schools were randomly selected from two Norwegian counties, Hedmark and Telemark, and all 6 th and 7 th graders in each school were invited to participate. All schools were public schools, as are most schools in Norway. Informed consent was sought from the children and their parents prior to the study. Ethical approval and research clearance was obtained from The National Committees for Research Ethics in Norway and from The Norwegian Social Science Data Services.

A survey questionnaire was completed by the pupils in the classroom in the presence of a trained project worker. One school-lesson (45 minutes) was used to complete the questionnaire. Out of 1065 eligible pupils, 896 completed the baseline questionnaire. A total of 816 (77%) also completed the follow-up questionnaire: 406 boys and 410 girls (444 6 th graders and 372 7 th graders). The average age of the sample at baseline was 11.8 years.

### Instrument

A questionnaire to measure the children's fruit and vegetable intake and potential predictors of intake was developed as part of the FVMM project. Repeated pre-testing, a test-retest study [11,12] and a validation study [11] of the questionnaire were conducted prior to the baseline survey.

The questionnaire items assessing potential predictors were guided by SCT. SCT postulates that behaviour (here fruit and vegetable intake) is a result of environmental and personal factors, but it also states that all three sets of factors affect each other in constant reciprocal relationships [2]. The following factors were measured:

Fruit and vegetable Intake was measured by four frequency questions: 'How often do you eat vegetables for dinner?,' 'How often do you eat other vegetables (e.g., carrot for school lunch)?' 'How often do you eat apple, orange, pear or banana?' and 'How often do you eat other fruits or berries?' All four questions had ten response alternatives ranging from 'Never' = 0 to 'Several times a day' = 10. These items were added together, and the test retest (14 days in-between) correlation of this scale in a sample of 114 6 th grade pupils was 0.75 [11]. The correlation between this scale and a validation method (7-day food diaries) was 0.32 in a separate validation study of 85 6 th grade pupils, which is similar to the results found in other studies of this age group [11].

A total of seven potentially mediating factors were measured; three environmental and four personal. All questionnaire items included in these seven scales are provided in Table 1, including response alternatives, scale ranges and psychometric properties (test retest correlation and internal consistency reliability [6,11,12]. The environmental factors were Accessibility at home, Accessibility at school and Modelling. The personal factors were Intention (to eat 5-a-day), Preferences, Self-Efficacy (to eat 5-a-day) and Awareness (of the 5-a-day recommendation). All scales except Awareness and Accessibility at school included one to five statements with response alternatives ranging from 'I fully disagree' to 'I fully agree.' Awareness (of the 5-a-day recommendation) was measured by one question: 'How many servings of fruit and vegetables should a person your age eat every day?' This question had seven response alternatives ranging from 'None' to 'More than 5 a day.' Accessibility at school was a dichotomous variable assessing whether the pupils subscribed to the Norwegian School Fruit Programme or not. As few Norwegian elementary schools have canteens, the only accessible fruits and vegetables in most Norwegian schools are through this programme. This programme offers pupils a piece of fruit or a carrot every day at the cost of NOK 2.50 per day (€ 0.30) [13]. All Norwegian elementary schools are offered the chance to participate in this programme. If the school participates, fruit and vegetables are available to the pupils, but it is not accessible to them unless they subscribe to the programme. As the Norwegian School Fruit Programme started in October 2001 in Hedmark and Telemark, no pupils subscribed at baseline (September 2001). Thus, the baseline score for all pupils was zero.

**Table 1:** Questionnaire items, response alternatives, and reliability coefficients (test-retest correlation and internal consistency reliability) of fruit and vegetable intake and the SCT variables assessed in the FVMM study.

**Scale**	**Response**	**Possible scale range**	Test-retest correlation (14 days in between)**	Internal consistency reliability***
Intake (times/week)		0/40	0.75	NA
How often do you eat: 1. Vegetables for dinner? 2. Vegetables on bread?* 3. Other vegetables (e.g. carrot for school lunch)? 4. Apple, orange, pear or banana? 5. Other fruits or berries?	Ten alternatives: Never (0), less than once a week (0.5), once a week (1) to every day (7), several times a day (10). Question 3 did not count in the scale.*			
Accessibility at home		-10/10	0.66	0.49
1. At home we usually always have fruits and vegetables in the refrigerator 2. At home I am allowed to eat fruits and vegetables whenever I want 3. Mother or father do sometimes cut up fruits or vegetables for me as a snack 4. At home we usually have vegetables at dinner every day 5. At home we usually have fruits available in a (fruit-) bowl	Five alternatives: I fully disagree (-2), I disagree (-1), I do not agree nor disagree (0), I agree (1), I fully agree (2)			
Accessibility at school		0/1	No data	NA
1. Do you subscribe to the School Fruit Programme?	Yes (1), no (0).			
Modelling		-8/8	0.70	0.46
1. My mother eats lots of fruits and vegetables 2. My father eats lots of fruits and vegetables 3. Many of my friends and siblings eat lots of fruits and vegetables 4. My home-economy teacher eats lots of fruits and vegetables	Five alternatives: I fully disagree (-2) to I fully agree (2)			
Intention (to eat 5-a-day)		-2/2	0.51	NA
1. I intend to eat at least 5 servings of fruits and vegetables every day	Five alternatives: I fully disagree (-2) to I fully agree (2)			
Preferences		-8/8	0.74	0.68
1. Fruits and vegetables make my meals taste better 2. I really like raw vegetables 3. Fruits are among the best [foods] I know 4. Fruits and vegetables are very suitable as snacks	Five alternatives: I fully disagree (-2) to I fully agree (2)			
Self-Efficacy (to eat 5-a-day)		-6/6	0.61	0.44
1. For me, it would be easy to eat fruits or vegetables to every meal, every day, if I decided to do so 2. For, me it would be easy to eat fruits or vegetables on Saturday evenings, even if everybody else are eating snacks 3. For me, it would be easy to eat more than 5 servings of fruits and vegetables every day	Five alternatives: I fully disagree (-2) to I fully agree (2)			
Awareness (of 5-a-day)		0/6	No data	NA
1. How many servings of fruit and vegetables should a person at your age eat every day?	Seven alternatives: None (0), 1-a-day (1) to 5-a-day(5), more than 5-a-day (6)			

* Some Norwegians have vegetables on their sandwiches, but usually in small amounts. Therefore, this question was not included in the intake scale. The question was included in the questionnaire so that the participants should not include their vegetables on bread in the 'other vegetables' question.

** Intake: Spearman's r From Andersen and colleagues [11], all other scales: Pearson's r from Bere and Klepp [12].

*** Cronbach's alpha: From Bere and colleagues [6].

NA = not applicable

### Statistics

Missing values on any item were substituted with the mean value for the remaining group on the respective item, if more than 50% of the scale items were answered. A total of 214 pupils had one or more missing values substituted. Multiple regression assumptions regarding normality, linearity and homoscedasticity were found to be acceptable, and therefore parametric statistics were used. Multiple regressions were performed to determine the explained variance of the children's fruit and vegetable intake and of the change in intake. Pearson's correlation coefficients (r) and standardized regression coefficients (beta) are given for each independent variable. In addition, the unique amount of variance in intake explained by an independent variable is given by the square of the semi-partial correlation (sri2) [14,15]. The square of the multiple correlation (= explained variance) is given by the multiple correlation (R2) and the adjusted multiple correlation (adj. R2).

The effect of potential interactions between baseline values of Preferences (dicotomised) and the change in Accessibility at home (positive or negative (including 0)), and baseline values of Accessibility at home (dicotomised) and change in Preferences (positive (including 0) or negative) and change in fruit and vegetable intake was assessed by including the respective cross-product terms into linear regression models. These models did also include the dicotomised change in Accessibility or the dicotomised change in Preferences respectively.

Paired sample t-tests were used in the attrition analyses. All analyses were conducted using SPSS version 12.

## Results

Mean values of intake and the SCT constructs at baseline and follow-up, as well as change scores are presented in Table 2.

**Table 2:** Baseline, follow-up and change mean scores and standard deviations (SD) of the variables assessed.

	**Baseline**			**Follow-up**			**Change**		
**Scale**	n	Mean	SD	n	Mean	SD	n	Mean	SD
Intake (times/week)	804	14.1	7.1	810	13.2	7.1	799	-0.9	6.6
Accessibility at home	815	3.8	3.6	813	4.2	3.5	812	0.5	3.6
Accessibility at school	816	0	NA	816	0.1	NA	816	0.1	NA
Modelling	796	2.0	2.7	791	2.0	2.7	774	-0.1	2.9
Intention (to eat 5-a-day)	809	0.2	1.3	812	0.1	1.3	805	-0.1	1.4
Preferences	810	2.7	3.8	813	2.1	3.9	807	-0.6	3.3
Self-Efficacy (to eat 5-a-day)	813	0.1	2.6	814	0.2	2.7	811	0.2	2.7
Awareness (of 5-a-day)	805	3.5	1.5	792	3.4	1.6	782	-0.1	1.8

NA = not applicable

### Correlates of intake cross-sectionally

Cross-sectionally, Accessibility at home and Preferences were most strongly correlated to intake (r = 0.43 and 0.45, respectively) (Table 3). At baseline, the independent variables explained 29% (adj. R2) of the variance in intake at baseline (Table 4, analysis I).

**Table 3:** Correlation (Pearson's r) between the SCT variables (baseline and change scores) and fruit and vegetable intake (baseline, follow-up and change scores).

	Fruit and vegetable intake		
	Baseline	Follow-up	Change
***Baseline:***			
Intake (= past intake)	1**	0.57**	NA
			
Accessibility at home	0.43**	0.31**	NA
Modelling	0.24**	0.18**	NA
Intention (to eat 5-a-day)	0.33**	0.27**	NA
Preferences	0.45**	0.35**	NA
Self-Efficacy (to eat 5-a-day)	0.35**	0.30**	NA
Awareness	0.22**	0.19**	NA
			
***Change in:***			
Accessibility at home	NA	0.13**	0.27**
Accessibility at school	NA	NA	NA
Modelling	NA	0.10**	0.11**
Intention (to eat 5-a-day)	NA	0.07*	0.14**
Preferences	NA	0.17**	0.28**
Self-Efficacy (to eat 5-a-day)	NA	0.12**	0.16**
Awareness	NA	0.11**	0.10**

NA = not applicable

* p < 0.05

** p < 0.01

**Table 4:** Multiple regressions of fruit and vegetable intake (baseline, follow-up and change) by the SCT variables (baseline and change) including the standardized regression coefficients (beta) and the semi-partial correlation (sri2).

	Fruit and vegetable intake									
	Baseline Analysis I (n = 766)		Analysis IIa (n = 770)		Follow-up Analysis IIb (n = 762)		Analysis IIc (n = 722)		Change Analysis III (n = 722)	
	beta	sri2	beta	sri2	beta	sri2	beta	sri2	beta	sri2
***Baseline***										
Accessibility at home	0.24**	0.04	0.15**	0.02	0.04	0.00				
Modelling	0.06	0.00	0.03	0.00	0.01	0.00				
Intention (to eat 5-a-day)	0.03	0.00	0.04	0.00	0.03	0.00				
Preferences	0.26**	0.04	0.18**	0.02	0.06	0.00				
Self-Efficacy (to eat 5-a-day)	0.08*	0.00	0.11**	0.01	0.07	0.00				
Awareness	0.09**	0.01	0.09*	0.01	0.05	0.00				
										
Past intake					0.47**	0.15	0.59**	0.34		
										
***Change in***										
Accessibility at home							0.14**	0.02	0.21**	0.04
Accessibility at school							0.17**	0.03	0.16**	0.03
Modelling							0.00	0.00	-0.02	0.00
Intention (to eat 5-a-day)							-0.03	0.00	-0.03	0.00
Preferences							0.17**	0.02	0.21**	0.03
Self-Efficacy (to eat 5-a-day)							0.05	0.00	0.05	0.00
Awareness							0.08**	0.01	0.07*	0.00
										
R2:	**0.30**		**0.18**		**0.33**		**0.44**		**0.16**	
Adj. R2:	**0.29**		**0.17**		**0.33**		**0.43**		**0.15**	
Sum sri2:		**0.10**		**0.05**		**0.16**		**0.42**		**0.11**

* p < 0.05

** p < 0.01

### Prediction of future intake

All independent variables (measured at baseline) were significantly correlated to future intake (measured at follow-up) (Table 3). These variables explained 17% of the variance in the pupils' fruit and vegetable intake at follow-up, with Modelling and Intention as the only non-significant variables (Table 4, analysis IIa). Overall, 5% (sum sri2) of the variance was explained by unique contribution to the explanation, while the remaining 12% was shared variance by two or more concepts. Accessibility at home and Preferences contributed most of the unique variance explained (explaining 2% each).

When reported fruit and vegetable intake at baseline (past intake) was included in the model, none of the other baseline variables remained significant (Table 4, analysis IIb). This model explained 33% of future fruit and vegetable intake. A model with past intake as the only independent variable explained 32% of the variance in future intake (data not shown).

In addition to past intake, the change in the independent variables explained 43% of the variance of follow-up intake, almost all by unique contribution by; past intake (35%), change in Accessibility at home (2%), change in Accessibility at school (3%), change in Preferences (2%) and change in Awareness (1%) (Table 4, analysis IIc).

### Correlates of change in intake

The changes in the independent variables were all significantly correlated to the change in intake (Table 3), and they explained 15% of the variance in the change in intake between baseline and follow-up, with Accessibility at home, Accessibility at school, Preferences and Awareness being significant (Table 4, analysis III). Overall, 11% of the variance was explained by unique contribution to the explanation. Accessibility at home, Accessibility at school and Preferences contributed most of the unique variance explained (4%, 3% and 3%, respectively).

### Interaction analyses

The cross-product of baseline Preferences and change in Accessibility at home was not significant when introduced in the model (p = 0.29). The cross-product of baseline Accessibility and change in Preferences was significant (p = 0.03), and therefore the relationship between change in Preferences and change in intake are presented in Table 5, stratified by baseline Accessibility at home. Table 5 shows that the difference in change in intake between those with positive and negative changes in Preferences was much greater among those with a high baseline Accessibility at home than those with a low accessibility (4.4 vs. 2.3 times/week).

**Table 5:** Changes in fruit and vegetable intake (times/week) related to changes in Preferences, stratified by baseline Accessibility at home

Baseline Accessibility at home	Change in Preferences	Change in intake	Confidence intervals
LOW (n = 424)	Negative (n = 188)	-1.7	(-2.6, -0.8)
	Positive (n = 230)	0.6	(-0.2, 1.4)
	p-value	< 0.01	
			
HIGH (n = 391)	Negative (n = 216)	-3.4	(-4.2, -2.5)
	Positive (n = 173)	1.0	(0.0, 1.9)
	p-value	< 0.01	

### Attrition analyses

No significant differences were seen between the cohort participants (n = 816) and the baseline-only participants (n = 80) for any of the variables assessed in this study. The pupils with scores on all scales assessed (n = 722, same sample as analyses IIc and III) had higher Preferences than those without follow-up data or without scores on all scales (n = 174, p = 0.05). Of the pupils with scores on all scales, those without missing data (n = 508) did not show different scores on any of the scales compared to pupils with one or more missing values replaced (N = 214).

## Discussion

These results from the FVMM project show that changes in Accessibility (at home and at school) and Preferences were correlated to changes in intake, and that these changes explained some of the variance of follow-up fruit and vegetable intake, when controlling for past intake. This suggests that these factors play a role as potential mediators in future intervention studies.

Prospectively, the change in SCT factors explained 15% of the change in intake, and together with past intake, 43% of the variance in future intake. We are not aware of any other studies assessing the prospective nature of fruit and vegetable predictors in children, and this is more explained variance than what has been reported for adults [16]. The present study contributes to the literature by showing that longitudinal relationships exist between accessibility, preferences and fruit and vegetable intake. Longitudinal relationships are necessary, but however, not a sufficient premise for causality.

While it is a prerequisite that fruits and vegetables are available and accessible, it is not necessarily sufficient to ensure high intake. A recent review of environmental interventions to promote fruit and vegetable consumption among youth in school settings reported only three stand-alone environmental interventions [4]. Only one of them was a stand-alone availability study, assessing the effect of a Danish pilot project of a school fruit and vegetable subscription programme [17]. The subscription programme increased 6–10 year old children's intake of fruit among both subscribing and non-subscribing pupils at the intervention schools, with about 0.4 pieces/school day, compared to control schools. The two other stand-alone environmental studies reported significant effects of lowering prices of fruit and vegetable on pupil purchases [18], and of a multi-environmental strategy [19]. In addition, a number of multi-component fruit and vegetable and multi-behavioural (including fruit and vegetables) interventions were included in this review [4]. These studies did not separately evaluate the availability/accessibility component, and unfortunately, the effect of that component cannot be stated. More recently, we have evaluated the effect of free participation in the Norwegian School Fruit Programme [13]. Seventh-graders at nine elementary schools were given a piece of fruit or a carrot every school day for a school year for free, and the pupils' mean intake of fruit and vegetables at school increased by about 0.9 portions compared to control pupils [13]. Offering free fruit at school can be seen as increasing the accessibility of fruit and vegetables at school, and this increased accessibility clearly led to increased intake. Increasing accessibility is theoretically simple; just offer children fruit and vegetables – at school or at home.

Food preferences have been suggested as determinants for food intake [20], including fruit and vegetable intake [6,8]. Previous research suggests that children's dislike of foods can be transformed into liking of foods with repeated tasting or 'exposure' to those foods [21,22]. It has also been reported that children's food preferences are often guided by taste alone, while food choices of adults also tend to be influenced by nutritional beliefs and attitudes toward weight and dieting [20]. However, a few studies have reported that children's preferences for and consumption of disliked vegetables were enhanced when children had opportunities to observe peers selecting and eating those foods, and that adults can also be effective in increasing fruit and vegetable intake by encouraging children to try new foods [23]. We are, however, not aware of any intervention studies that have increased children's or adolescents' fruit and vegetable intake through increased preferences.

In the present study we also found an interaction between baseline accessibility at home and the relationship between change in preferences and change in intake, indicating that baseline accessibility mediate this relationship. For those with high baseline accessibility, changes in preferences were related to significantly larger changes in intake than for those with low baseline accessibility, indicating again that high access to fruits and vegetables are extremely important for a sufficient fruit and vegetable intake. This result is in line with previously cross-sectionally reported interactions between accessibility and preferences. Neumark-Stainer et al. [8] found that, in a group of adolescents (mean age 14.9 years), preferences was more related to intake for those with higher levels of accessibility. For those with the lowest accessibility, preferences were not related to intake. Similarly, Cullen and colleagues [7] found in a group of 4–6 graders that among those with low preferences, both availability and accessibility were significant in explaining the variance in fruit and vegetable intake. For those with high preferences, only availability was significant. This again indicates that those with lower preferences need a higher access to fruit and vegetables in order to eat sufficient amounts of fruit and vegetables.

In addition to changes in Accessibility and Preferences, change in Awareness of the 5-a-day recommendations contributed significantly to the explanation of the variance in future fruit and vegetable intake. A change in Awareness also contributed significantly to the explanation of variance in the change in intake. Recently, Reynolds and colleagues [24] showed that a similar scale was a significant mediator in the *High 5 Alabama *intervention study (an increase in Awareness explained 9.8% of the increase observed in fruit and vegetable intake). It has also been reported from several countries that several people are not aware of national fruit and vegetable recommendations [25-28]. Thus, our results are encouraging, and relevant information about existing 5-a-day recommendations should be included in future intervention studies.

The strength of this study is that it includes a prospective cohort of a rather large random sample of schools. There are, however, also some limitations with the present study. The study was geographically confined to two of Norway's 19 counties. As Norway is a rather homogeneous country, we believe the results are likely to be generalizable to the other counties. A second limitation is the validity of the intake measure as this scale showed a rather low correlation with the validation method [11]. However, the correlation was not lower than found in other studies of same age pupils, and the scale showed good test-retest reliability. A third limitation is that the follow-up period was only 8–9 months. In such a short time span, large changes in fruit and vegetable intake can not be expected. A small change in intake will be a limiting factor for observing relations between change in intake and its determinants. However, due to an age-related decline in fruit and vegetable intake previously observed in Norway [9] and elsewhere in Europe [29], as well as seasonal variations in Norway, the average change in intake was -0.9 times/week (Table 2). Finally, when using observational data, prospective relationships can, as for cross-sectional studies, be due to a third antecedent. Thus we can still not state causality.

## Conclusion

Changes in Accessibility and Preferences and Awareness were significantly correlated to changes in reported fruit and vegetable intake, and as hypothesised, these changes also explained added variance in future fruit and vegetable intake when adjusting for past intake. Baseline accessibility was a moderator of the relationship between change in preferences and change in intake. These results point to the potential role of these factors, especially accessibility, as mediators in future fruit and vegetable interventions.

## Competing interests

The author(s) declare that they have no competing interests.

## Authors' contributions

EB collected and analysed the data and drafted the manuscript. KIK conceived the study, participated in its design and coordination, and provided critical revision of the paper. Both authors have read and approved the final manuscript.
